# Influence of antitox and Vitamin E-selenium on meat quality and safety in rabbits after 1,1-experimental dimethylhydrazine toxicosis

**DOI:** 10.14202/vetworld.2020.1567-1572

**Published:** 2020-08-12

**Authors:** Balgabay S. Maikanov, Mikhail V. Zabolotnykh, Laura T. Auteleyeva, Symbat P. Seidenova

**Affiliations:** 1 Faculty of Veterinary Sciences & Animal Husbandry, Department of Veterinary Sanitation, Saken Seifullin Kazakh Agrotechnical University, Zhenis Avenue, 62, Nur-Sultan, 010000, Kazakhstan; 2 Institute of Veterinary Medicine and Biotechnologies, Omsk State Agrarian University named after P.A. Stolypin, October Street, 92, Omsk, 644122, Russian Federation

**Keywords:** 1,1-dimethylhydrazine, amino acid composition, detoxification, detoxifying mixture, meat, rabbits

## Abstract

**Aim:**

This study investigated the effects of antitox and Vitamin E-selenium on meat quality and safety in rabbits after experimental 1,1-dimethylhydrazine (1,1-DMH) toxicosis.

**Materials and Methods:**

Experimental groups of rabbits weighing 1.5-2.5 kg each were kept in a vivarium under same controlled conditions (temperature 16-21°C, humidity 60-80%, 12/12 h light/dark cycle, noise level <85 dB) with free access to standard food containing 22.0% protein, 4.5% fat, and 4% fiber. The effect of a detoxifying mixture of antitox and Vitamin E-selenium on safety indicators (residual amount of 1,1-DMH) and quality indicators pre- and post-detoxification of the rabbits from 1,1-DMH was determined.

**Results:**

After detoxification, the residual 1,1-DMH level decreased in all organs and tissues to <0.1 mg/kg. The nutritional value of meat increased by improving organoleptic, physical, and chemical parameters and the amino acid composition of protein.

**Conclusion:**

The antitox+Vitamin E-selenium detoxifying mixture significantly decreases the residual 1,1-DMH level in organs and tissues of animals and increases the nutritional value of rabbit meat in subacute poisoning. The detoxifying mixture can also be used on animals grazing in territories adjacent to Proton-M launch sites for preventive and therapeutic purposes.

## Introduction

Ecological system contamination by mutagenic xenobiotics has attracted increasingly attention of researchers. One type of chemical pollution of ecosystems is caused by the fall of separable steps of rockets containing residues of 1,1-dimethylhydrazine (1,1-DMH) fuel [1]. 1,1-DMH is an unstable, highly toxic hazard Class 1 chemical. Therefore, it is easily oxidized [2].

1,1-DMH has a toxic and skin-irritating effect on both humans and animals. It can enter the body through the respiratory, integumentary, or gastrointestinal system. In the body, 1,1-DMH is distributed evenly, affecting the liver, central nervous system (CNS), cardiovascular system, and hematopoietic system [3,4].

So far, seven groups of neutralization methods have been tested under laboratory and industrial conditions: Chemical, biochemical oxidation, radiation, thermal, catalytic neutralization, vapor absorption, and leakage dilution [5].

Studies at the Baikonur Cosmodrome, Kazakhstan, have reported the use of potassium permanganate (sodium or calcium) with promoters as an effective method of neutralizing leakages at unsymmetrical 1,1-DMH (UDMH) concentrations of up to 0.1% [6]. The Baikonur Cosmodrome has played a significant part in the history of rocket science and cosmonautics. The first intercontinental ballistic missile R-7 was successfully launched from here in August 1957 and reached the Kamchatka Peninsula at a distance of 6000 km. Six weeks later, the modified R-7 launched the first artificial Earth satellite into orbit. In 1961, the first cosmonaut, Yuri Gagarin, started his space trip from the same launch pad [7].

One method of partial neutralization is to treat UDMH leakage with mineral and organic acid solutions. For this, nitric, hydrochloric, sulfuric perchloric, acetic, oxalic, and phosphoric acids are used [8]. However, there are no effective methods of neutralizing 1,1-DMH and its decay products; the duration of soil self-cleaning from 1,1-DMH is up to 34 years and from kerosene 5 years. During the period of the Baikonur Cosmodrome operation (1957-2003), the total volume of atomized 1,1-DMH was ~2000 tons [9].

For detoxification of 1,1-DMH in atmospheric air, sorption by lignohumic substances for disinfection of rocket fuel leakage is recommended (Patent 2529999). The sorbent is hydrolyzed by humification *in vivo* lignin and absorbs UDMH at high speed by strong binding of 1,1-DMH due to chemical interactions with the functional groups of lignin and humic substances [10].

In the case of CNS and liver damage caused by UDMH, curcumin has a protective effect [11,12]. Researchers in India induced adenomas and colon carcinomas in rats by introducing DMH, and they found that administration of vanadium salt not only increased glutathione S-transferase and cytochrome P450 activity in the liver but also slowed tumor growth [13].

Because of the use of hazard Class 1 and 2 chemicals while preparing and starting launch vehicles and spacecraft, environmental safety is a major issue in the space-rocket industry. The greatest environmental risks arise with 1,1-DMH turnover, which is used as a component of rocket fuel in Proton class launch vehicles [14].

The main environmental impact of launches is caused by the fall of separable steps of launch vehicles. The separation of the first step of Proton-M occurs in the Ulytau district of the Karaganda region of Kazakhstan. In territories with fresh falls and UDMH leakage, air pollution is also observed [15].

UDMH in the soil can migrate along with the soil profile into groundwater and open water spaces, accumulate in herbs and cultivated plants, and then enter the bodies of animals and humans. Numerous studies have confirmed the existing potential danger of the transmission of rocket fuel components along the soil-plant-animal-human food chain. The introduction of these substances into the bodies of animals causes intoxication or disease, decreasing in the quality of livestock products.

Antitox (1 mL) comprises 0.2 g of sodium thiosulfate, 0.022 g of sodium glutamate, 0.001 g of chlorkresol, and 0.0005 g of sodium bisulfite. The drug is used to detoxify the body by binding with toxic products to form nontoxic sulfites and remove toxic substances from the body [16]. It has a stabilizing effect on hepatocyte membranes; improves their energy supply; normalizes protein, carbohydrate, and fat metabolism; and increases the body’s resistance to hypoxia. Vitamin E-selenium makes up for the lack of Vitamin E and selenium in animals [17]. The biological role of selenium is related to its antioxidant properties. Selenium helps remove toxic substances from the body and increase immunity. The drug leads to a rapid increase in Vitamin E and selenium levels and normalizes metabolic processes [18].

This study investigated the effects of antitox and Vitamin E-selenium on the quality and safety of rabbit meat after experimental 1,1-DMH toxicosis.

## Materials and Methods

### Ethical approval

The study was approved by the Ethics Commission of the Faculty of Veterinary Medicine and Animal Husbandry Technology of S. Seifullin NAO KATU (extract from #1 protocol dated 02.02.2017).

### Materials

The research material included samples of rabbit meat and internal organs. Experimental 1,1-DMH toxicosis was induced by per os administration of 0.5 mg/kg (subacute poisoning) of 1,1-DMH for 30 days. 1,1-DMH, 98% GSO (state standard sample), was obtained from Sigma-Aldrich (Germany). To work with 1,1-DMH (GSO), we took a special course “Industrial Safety at Hazardous Production Facilities,” granting the qualification of “Personnel authorized to work with potent toxic substances and hazardous substances.”

### Study period and location

The study was carried out from September 2018 to December 2019. The study was carried out in the vivarium of the Veterinary Clinic and in the Food Safety Laboratory of the Kazakh Agro Technical University names after Seifullin S. Additional study was carried out at the Republican State Enterprise “Graysh Ecology “Research Center” or the Ministry of Digital Development, Innovation and Aerospace Industry of the Republic of Kazakhstan Aerospace Committee as well as in the Laboratory of Biochemistry of the Siberian Research and Design Technological Institute of Animal Breeding of the Siberian Federal Scientific Center of Agrobiotechnology of the Russian Academy Sciences, Novosibirsk (Russian Federation).

### Experimental design

The design of the experiments was that the detoxifier was tested on a group of experimental animals. The animals were pre-etched with 1,1 dimethylhydrazine in the dose we selected (Table-1).

**Table-1:** Experiment design.

Sr. No.	Group	Dose 1, 1-DMG mg/kg/day	Detoxifying mixture	Studied indicators
1.	Experimental #1 (n=5)	0.5/30	-	Residual amount of 1,1-DMG, organoleptic and physicochemical indicators, amino acid composition
2.	Experimental #2 (n=5)	0.5/30	Antitox 3 ml, i/m - E-Selen 0.04 ml/1 kg 10 days*	
3.	Control (n=5)	-	-	Organoleptic and physicochemical indicators, amino acid composition

### Methods

The mass fraction of 1,1-DMH in muscle tissue samples in the range of 0.1-20.0 mg/kg was measured by ion chromatography with amperometric detection (RSI SIC; Karysh-Ecology, Zhezkazgan, Kazakhstan). The technique is based on UDMH extraction from muscle tissue into the aqueous phase by sample homogenization, protein separation by precipitation and centrifugation, and centrifugate analysis by ion chromatography with amperometric detection. The UDMH peak area was proportional to its concentration, and the proportionality coefficient was set during chromatograph calibration. A liquid and ion chromatograph, analytical small size, color Yauza (MEKV. 414538.001 PS), with an electrochemical detector, complete with the type PCIBMAT PC, and the corresponding software were used [19].

Amino acids in tissues were detected according to GOST 32195-2013, “Method for Determination of Amino Acid Content,” using a Shimadzu LC-20 Prominence liquid chromatograph (Shimadzu Corporation, Kyoto, Japan) with a fluorimetric and spectrophotometric detector. Standard samples of amino acids (Sigma-Aldrich), acetonitrile *puriss. spec*., isopropyl alcohol *puriss. spec*., fluorescein isothiocyanate (FTIC; Sigma-Aldrich), phosphoric acid, and sodium acetate were used. In addition, a 25 cm×4.6 mm SUPELCO C18, 5 μm chromatography column measuring (Supelco Analytical, Bellefonte, PA, USA) with a precolumn was used to protect the main column from impurities.

Organoleptic studies to observe the appearance, texture, transparency, and smell of the broth, as well as sample preparation, were performed according to GOST 51477-99 “Meat and Meat Products. Sampling Methods.” Physical and chemical parameters were analyzed using reactions to peroxidase and pH with copper sulfate and Nessler’s reagent.

### Statistical analysis

Statistical analysis of results was performed using Student’s t-test. p<0.05 was considered statistically significant.

## Results and Discussion

**Figure-1:**
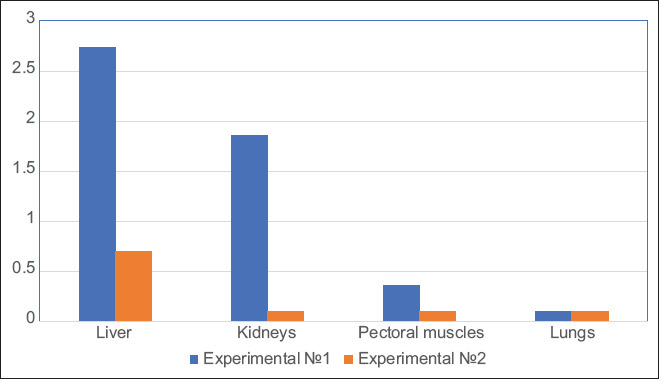
The concentration of 1,1-dimethylhydrazine n the organs and tissues of rabbits before and after detoxification.

Because of subacute exposure, we found a residual 1,1-DMH level in all edible parts of experimental Group 1. The degree of 1,1-DMH accumulation in descending order was as follows: Liver, 2.74±0.82 mg/kg; kidneys, 1.86±0.56 mg/kg; pectoral muscles, 0.36±0.11 mg/kg; and lungs, <0.1 mg/kg. We also observed an excess (0.002 mg/kg) of the maximum permissible indicators (Table-2 and Figure-1). The data were correlated with the previous studies that reported the carcinogenic effects of 1,1-DMH and its derivatives on the structure and function of the liver [20]. Hydrazine and its derivatives are evenly distributed over organs and tissues, and their highest quantity is found in the kidneys and liver [21].

**Table-2:** The concentration of 1,1-dimethylhydrazine in the organs and tissues of rabbits before and after detoxification.

Sr. No.	Organ	Experimental No.1	Experimental No.2
1.	Liver	2.74±0.82	0.70±0.21
2.	Kidneys	1.86±0.56	<0.1
3.	Pectoral muscles	0.36 ±0.11	<0.1
4.	Lungs	<0.1	<0.1

p≤0.01

Studies have reported only sporadic information about the genotoxic effects of 1,1-DMH and its derivatives and their effects on functions of the CNS, cardiovascular system, and lymphatic system and on histohematological barrier permeability. The methods of neutralizing 1,1-DMH and its derivatives in humans, animals, and plants have not been sufficiently studied [22,23]. 1,1-DMH has the highest possible carcinogenic effect, inducing liver tumor in 100% of animals [24]. According to the US Department of Healthcare, the International Agency on Cancer Research, and the World Health Organization, hydrazine and its derivatives are potential carcinogens [25].

On the basis of the results of a patent search and reconnaissance experiments, we selected a detoxifying mixture of antitox and Vitamin E-selenium. After applying an intramuscular injection of antitox and Vitamin E-selenium on a daily basis to experimental Group 2, the 1,1-DMH level decreased from 2.74±0.82 to 0.70±0.21 mg/kg in the liver, from 1.86±0.56 to <0.1 mg/kg in the kidneys, and from 0.36±0.11 to <0.1 mg/kg in the pectoral muscles (p≤0.01).

Antitox removes toxic substances from the body and is recommended in cases of animal poisoning and for the treatment of inflammation and metabolic disorders [26].

As mentioned earlier, selenium helps eliminate toxic substances from the body and increases immunity. Its biological role is associated with its antioxidant properties. Administration of selenium leads to a rapid increase in the Vitamin E and selenium content in the body and normalizes metabolic processes [18].

Organoleptic and physicochemical studies showed that experimental Group 1 was sick compared to the control group. Significant deviations were observed in the sampling by cooking: The broth was cloudy and had an unpleasant smell, muscles in the incision were flabby, moist, and not dense and left a wet spot on paper, and the smell ranged from sourish to sour. The pH ranged from 6.2±0.12 to 6.34±0.24. Positive results in determining the presence of primary proteolysis of all samples being studied indicates the presence of free amino acids and ammonium salts.

Experimental Group 1, therefore, had the clinical status of “sick animals” before detoxification, as shown by pH indicators. The action of the toxin suppresses the enzymatic activity of the liver [27].

Post-treatment with antitox and Vitamin E-selenium, experimental Group 2 showed normal organoleptic and physicochemical parameters compared to experimental Group 1. The average pH decreased to 5.9±0.13. Negative results in determining the presence of primary proteolysis of all samples being studied indicates the absence of free amino acids and ammonium salts (Table-3) (p≤0.01).

**Table-3:** Physicochemical characteristics of rabbit meat.

Sr. No.	Indicator	Group		

		Experimental #1	Experimental #2	Control
1.	Peroxidase response	−	+	+
2.	−	6.3±0.24	5.9±0.13	5.9±0.09
3.	The reaction with a 5% solution of copper sulfate	Seeming clouding of the extract	Transparent	Transparent
4.	Identification of ammonia and ammonium salts	Yellow color with precipitation	Intense yellow color, slight clouded	Transparent

p≤0.01

The amino acid composition characterizes the nutritional and biological value of meat. Our antitox and Vitamin E-selenium detoxifying mixture improved the nutritional value of meat in experimental Group 2 because of an increase in the amino acid composition: Total amount of amino acids, number of individual essential, and non-essential acids, ratio of the sum of irreplaceable and replaceable amino acids, and amino acid rate.

In experimental Group 2, the qualitative and quantitative structure of the composition of all amino acids of meat changed (Table-4).

**Table-4:** Amino acid composition of the muscle protein of rabbit meat (%).

Sr. No.	Amino acid	Experimental group 1	Experimental group 2
Irreplaceable			
1.	Lysine	1.22±0.03	1.33±0.02 ↑
2.	Leucine	1.40±0.01	1.42±0.01 ↑
3.	Isoleucine	0.96±0.005	1.02±0.01
4.	Methionine	0.41±0.01	0.46±0.004
5.	Cystine	0.23±0.01	0.28±0.02
6.	Phenylalanine	0.66±0.07	0.69±0.01
7.	Tyrosine	0.90±0.02	1.00±0.004 ↑
8.	Threonine	0.93±0.01	0.89±0.05
9.	Tryptophan	0.84±0.03	0.30±0.05 ↓
10.	Valine	0.22±0.02	0.88±0.01 ↑
Total		7.77	8.27
Replaceable			
1.	Asparagine	0.78±0.01	1.82±0.002 ↑
2.	Serine	1.50±0.01	0.77±0.02 ↓
3.	Glutamine	0.78±0.1	2.68±0.03 ↑
4.	Glycine	2.54±0.01	1.14±0.02
5.	Alanine	0.88±0.02	1.27±0.03 ↑
6.	Arginine	1.09±0.05	1.16±0.01
7.	Histidine	0.73±0.1	0.79±0.01
8.	Proline	0.59±0.01	0.67±0.02
9.	Oxyproline	0.07±0.2	0.07±0.01
Total		8.96	10.37

p≤0.05

We established a verified increase in some irreplaceable and replaceable amino acids: Isoleucine increased to 1.02±0.01 (by 5.8%), lysine to 1.33±0.02 (by 8.2%), tyrosine to 1.00±0.004 (by 10%), valine up to 0.88±0.01 (by 75%), alanine to 1.27±0.03 (by 30%), aspartic acid to 1.82±0.002 (by 57.1%), and glutamic acid to 2.68±0.03 (by 70.9%) (p≤0.05).

The total amount of amino acids in the protein of meat of experimental Group 2 increased by 10.2% and the replaceable-to-irreplaceable amino acid ratio was 0.79 (Table-5).

**Table-5:** Amino acid indicators.

Sr. No.	Parameters	Experimental group 1	Experimental group 2
1.	Total amount of amino acids	16.73	18.64
2.	The ratio of the amount of replaceable to irreplaceable	0.86	0.79

The main indicator of the biological value of protein is the amino acid rate [28]. The limiting amino acids before and after detoxification were methionine (55.5%-62%), valine (20.85%-69.1%), cystine (31.14%-37.7%), phenylalanine (52.13%-54.5%), and methionine (55.5%). The amino acid rate of rabbit meat rises after detoxification, improving its biological and nutritional value (Table-6) (p≤0.05).

**Table-6:** Amino acid rate of meat protein of the rabbits of experimental groups, %.

Sr. No.	Amino acid	Experimental group 1	Experimental group 2
1.	Lysine	105.12	114.6
2.	Leucine	94.7	96.1
3.	Isoleucine	113.7	120.8
4.	Methionine	55.5	62.2
5.	Cystine	31.14	37.71
6.	Phenylalanine	52.13	54.50
7.	Tyrosine	71.08	78.9
8	Threonine	110.18	105.4
9.	Tryptophan	398.1	142.1
10.	Valine	20.85	69.1

p≤0.05

Many studies have reported the detoxification of 1,1-DMH and its derivatives in animals. There is evidence of the participation of antioxidant defense systems, especially the glutathione system, in the detoxification of hydrazine and its derivatives. In addition, studies have reported the effect of hydrazine and its derivatives on free radical oxidation reactions and the decreased glutathione level in cells of experimental animals [29]. A comprehensive study on histological preparations and physiological parameters of the body showed a significant decrease in toxic phenomena after the drug salsocollin was administered: In all groups, destructive changes in the liver, kidneys, and brain were replaced by regenerative processes [30]. Yaguzhinsky found an effective preventive and therapeutic agent that decreases the toxic effects of 1,1-DMH [31]. This is an analog pyridoxal phosphate Vitamin B6 with the trade name Pyridoxine. Acetone and acetaldehyde also partially weaken the effect of 1,1-DMH [31]. Researchers have proposed an approach to identifying biologically active substances that effectively decrease the effect of low doses of 1,1-DMH. Studies have also reported prospects of using biologically active additives based on curcumin (0.048 mcg/mL concentration) and on a combination of general and curcumin compounds in a 1:1 ratio (0.017 mcg/mL) for the protection of people and personnel [32].

Our antitox and Vitamin E-selenium detoxifying mixture not only decreases the level of toxic action of 1,1-DMH in organs and tissues but also significantly increases the amino acid composition of rabbit meat protein.

## Conclusion

The antitox and Vitamin E-selenium detoxifying mixture significantly decreases the 1,1-DMH level in the organs and tissues of animals and increases the nutritional value of rabbit meat. This detoxifying mixture can also protect livestock from subacute 1,1-DMH poisoning.

## Authors’ Contributions

BSM and MVZ: Conception and design, analysis and interpretation of data. LTA: Acquisition of data and drafting of the manuscript. SPS: Analysis and interpretation of data and critical revision. All authors read and approved the final manuscript.

## Competing Interests

The authors declare that they have no competing interests.

## Publisher’s Note

Veterinary World remains neutral with regard to jurisdictional claims in published institutional affiliation.
